# Lymph node volume predicts survival but not nodal clearance in Stage IIIA-IIIB NSCLC

**DOI:** 10.1371/journal.pone.0174268

**Published:** 2017-04-20

**Authors:** Vishesh Agrawal, Thibaud P. Coroller, Ying Hou, Stephanie W. Lee, John L. Romano, Elizabeth H. Baldini, Aileen B. Chen, David Kozono, Scott J. Swanson, Jon O. Wee, Hugo J. W. L. Aerts, Raymond H. Mak

**Affiliations:** 1 Department of Radiation Oncology, Dana-Farber Cancer Institute, Brigham and Women's Hospital, Boston, MA, United States of America; 2 Harvard Medical School, Boston, MA, United States of America; 3 Division of Thoracic Surgery, Brigham and Women's Hospital, Boston, MA, United States of America; 4 Department of Radiology, Brigham and Women's Hospital, Boston, MA, United States of America; Baylor College of Medicine, UNITED STATES

## Abstract

**Background:**

Locally advanced non-small cell lung cancer (LA-NSCLC) patients have poorer survival and local control with mediastinal node (N2) tumor involvement at resection. Earlier assessment of nodal burden could inform clinical decision-making prior to surgery. This study evaluated the association between clinical outcomes and lymph node volume before and after neoadjuvant therapy.

**Materials and methods:**

CT imaging of patients with operable LA-NSCLC treated with chemoradiation and surgical resection was assessed. Clinically involved lymph node stations were identified by FDG-PET or mediastinoscopy. Locoregional recurrence (LRR), distant metastasis (DM), progression free survival (PFS) and overall survival (OS) were analyzed by the Kaplan Meier method, concordance index and Cox regression.

**Results:**

73 patients with Stage IIIA-IIIB NSCLC treated with neoadjuvant chemoradiation and surgical resection were identified. The median RT dose was 54 Gy and all patients received concurrent chemotherapy. Involved lymph node volume was significantly associated with LRR and OS but not DM on univariate analysis. Additionally, lymph node volume greater than 10.6 cm^3^ after the completion of preoperative chemoradiation was associated with increased LRR (p<0.001) and decreased OS (p = 0.04). There was no association between nodal volumes and nodal clearance.

**Conclusion:**

For patients with LA-NSCLC, large volume nodal disease post-chemoradiation is associated with increased risk of locoregional recurrence and decreased survival. Nodal volume can thus be used to further stratify patients within the heterogeneous Stage IIIA-IIIB population and potentially guide clinical decision-making.

## Introduction

Lung cancer accounts for the greatest number of cancer-related deaths in the United States and has a 5 year survival rate of only 18% [1]. Lung cancer remains a challenging disease to treat, particularly for patients with advanced disease. Patients with locally advanced non–small-cell lung cancer (LA NSCLC) are composed of heterogeneous Stage III patients who are candidates for therapy with chemotherapy, radiation therapy and/or surgical resection [2]. LA NSCLC patients with mediastinal nodal involvement (N2) have decreased overall survival and local control compared to patients with hilar/peribronchial nodal disease (N1) or no nodal involvement (N0) [3]. The SWOG 8805 trial demonstrated that the strongest predictor of survival for patients with LA NSCLC treated with chemoradiation followed by resection was complete nodal clearance (defined as no evidence of disease in lymph nodes at the time of resection) [4]. Subsequent studies further validated the association between increased survival rates and patients who achieve mediastinal nodal clearance or downstaging following chemoradiation and surgical resection [5–10].

Despite substantial data that the eradication of tumor from mediastinal lymph nodes is strongly associated with overall survival, the prognostic value of lymph node burden both at initial diagnosis and after induction chemoradiation is less clear [11–14]. Alexander et al reported a significant association between increased lymph node volume and decreased survival [15], however Dehing-Oberije et al demonstrated a lack of association between lymph node size and survival [13]. Basaki et al demonstrated that lymph node volume is not associated with survival in stage III patients however their cohort was limited to patients undergoing definitive chemoradiation without surgical resection [16]. As a result, the prognostic value of lymph node size is difficult to ascertain from existing literature due to the heterogeneity of NSCLC stages and treatment modalities studied. A better understanding of the nodal component of staging can further stratify patients into high or low risk disease categories beyond existing known prognostic factors such as performance status [17], number of involved nodal stations [18], and stage [19].

Given the heterogeneity of presentations of LA NSCLC, the aim of this study was to identify locally advanced (Stage IIIA and IIIB) patients with greater risk of local recurrence or decreased survival based on extent of lymph node involvement prior to the initiation of preoperative chemoradiation as well as after completion of therapy. Additionally, we determine if these factors were predictive for histopathological nodal downstaging as this could potentially be a surrogate endpoint for survival.

## Methods

### Patient selection

The study was conducted under an IRB approved protocol. The research was conducted under a Dana-Farber /Harvard Cancer Center IRB approved protocol with a waiver of consent (due to retrospective nature of the study). We identified 209 patients with Stage IIIA-IIIB NSCLC (AJCC 7^th^ edition) treated between 2003–2013 with chemoradiation followed by surgical resection. Patients without available treatment plans from the treating radiation oncologist were excluded. To maintain consistent image quality, patients with CT imaging slice thickness greater than 5.0 mm were excluded from this study. Of the remaining patients, 93 patients had computed tomography (CT) imaging available after completion of radiotherapy prior to surgical resection. Of these, 73 patients had one or more clinically positive mediastinal (N2) lymph node. Clinically positive lymph nodes were defined by staging CT > 1 cm in short axis or positron emission tomography (PET) with SUV max > 3. Staging PET scans were performed for 70 patients. Pathologic confirmation of mediastinal lymph nodes was performed by cervical mediastinoscopy in 62 patients, including the three patients who did not receive a staging PET scan.

### Tumor segmentation and size calculations

Tumor segmentation was performed initially by the treating radiation oncologist. Two graduate students and one physician (V.A., T.C., Y.H.) subsequently modified existing gross tumor volume (GTV), internal target volume (ITV) or clinical target volume (CTV) contours to exclude air, blood vessels, or other normal tissue. These volumes were subsequently verified by an expert thoracic radiation oncologist (R.H.M). All volumes and diameters were calculated using MIM Maestro (MIM Software Inc., Cleveland, OH). Uni-dimensional diameters were measured according to RECIST 1.1 guidelines [20].

### Outcomes

Locoregional recurrence (LRR) was defined as recurrence at the resection site, hilar nodes, mediastinal nodes, or supraclavicular nodes. All other sites of recurrence were defined as distant recurrences (DR). Time to LRR and DR was defined as the interval from date of surgery to the first radiographically evident LRR and DR, respectively, and censored at the date of last negative re-staging scans in patients without recurrence or patients who died without recurrence. PFS was defined as the time from the date of surgery until any recurrence or death from any cause, and censored at the last date of follow-up. OS was defined as the time from the date of surgery until death from any cause, and censored at the last date of follow-up. Pathologic staging (yp) was determined at the time of surgical resection from institutional pathology reports. Complete tumor eradication from lymph nodes was considered ypN0. Nodal downstaging was defined as all clinical N2 or N3 disease with pathologic staging at the time of surgery of ypN0 or ypN1. Nodal clearance was defined as all clinical N2 or N3 disease with pathologic staging of ypN0 at the time of surgery.

### Statistical analysis

All statistical analysis was performed using SAS ver 9.4 (SAS Institute, Cary, NC) and R software version 3.2.2. Logistic regression was used to determine univariate relationship between continuous imaging variables and categorical outcome of nodal downstaging or nodal clearance. P-values generated for imaging characteristics during univariate analysis were corrected for multiple testing the false discovery rate (FDR) method of Benjamini and Hochberg [21]. Concordance index, cox regression, and Kaplan Meier analysis and log-rank test were used to determine significance of clinical variables and time-dependent outcomes. Concordance index and comparisons between concordance indices were calculated using the R survcomp package version 1.16 from Bioconductor. Lasso variable selection and cross-validation were performed using the R glmnet package version 2.0. P-values less than 0.05 were considered significant.

## Results

### Baseline patient characteristics

As shown in Table 1, among the 73 patients, the median age was 60 years (range 32–75) and the cohort was predominantly Caucasian (90.4%) and female (69.9%). There was a majority of Stage IIIA patients (83.6%) with adenocarcinoma as the predominant histology (65.8%). Sixty-six patients had N2 disease at the time of presentation and 7 patients had N3 disease. All patients received concurrent chemotherapy with the majority receiving cisplatin and etoposide (69.9%). All patients were treated with intensity-modulated radiation therapy (IMRT) (13.7%) or 3-dimensional conformal radiation therapy (3D CRT) (86.3%). Surgical resection consisted of wedge/sublobar resection (13.7%), lobectomy (78.1%), or pneumonectomy (8.2%).

**Table 1:** Patient and treatment characteristics of locally advanced NSCLC patients reported as number of patients (% of total patients).

Patient Characteristics (n = 73)	n (%)
Age (yr)	
Median (Range)	60 (32–75)
Q1-Q3	53–65
Gender	
Male	22 (30.1)
Female	51 (69.9)
Race	
White	66 (90.4)
Other (African American, Hispanic, Asian)	7 (9.6)
ECOG Performance Status	
0	30 (41.1)
1	39 (53.4)
2	4 (5.5)
AJCC Stage	
IIIA	61 (83.6)
IIIB	12 (16.4)
T Stage	
T1	18 (24.7)
T2	32 (43.8)
T3	17 (23.3)
T4	6 (8.2)
N Stage	
N2	66 (90.4)
N3	7 (9.6)
NSCLC Histology	
Adenocarcinoma	48 (65.8)
Squamous cell carcinoma	16 (21.9)
Other	9 (12.3)
**Treatment Characteristics**	
Chemotherapy	
Induction + concurrent	9 (12.3)
Concurrent	37 (50.7)
Concurrent + Adjuvant	27 (37.0)
Concurrent Chemotherapy	
Weekly carboplatin + taxol	17 (23.3)
Cisplatin + etoposide (EP 50/50)	51 (69.9)
Other	5 (6.8)
Surgery	
Lobectomy/Bilobectomy	57 (78.1)
Pneumonectomy	6 (8.2)
Wedge resection or sublobar resection	10 (13.7)
Radiation Technique	
3DCRT	63 (86.3)
IMRT	10 (13.7)
RT Dose	
54 Gy	49 (67.1)
55–60 Gy	8 (11.0)
≥ 66 Gy	16 (21.9)

### Treatment outcomes

Patient outcomes are shown in **Table 2**. Median follow up was 36 months, (range 0.4–113 months). Median OS was 78 months, and median time to DR was 68.6 months. The median time to LRR endpoint was not reached. The 3-year estimates of LRR, DM, PFS and OS were 28%, 38%, 51% and 68% respectively.

**Table 2:** Treatment outcomes reported at follow up intervals of 1 and 3 years following surgical resection.

Treatment Outcomes	Median (months)	1 year	3 year
Follow up	36		
Overall survival	78	85%	68%
Progression-free survival	36	79%	51%
Distant metastasis	68.6	23%	38%
Locoregional recurrence	NR*	12%	28%

*NR: endpoint not-reached

### Lymph node stations and volume predict LRR, PFS and OS

The median number of clinically positive lymph node stations was 3 (Range 1–8) and the median number of clinically positive N2 nodal stations was 2 (Range 1–6). Including the primary tumor and all involved nodal stations, the median tumor volume prior to chemoradiation was 39.1 cm^3^ (IQR 20.6 to 80.7 cm^3^). Median lymph node volume was 10.3 cm^3^ (IQR range 4.7 to 21.0 cm^3^). Of the total lymph node volume, the median N2 lymph node volume prior to chemoradiation was 6.7 cm^3^ (IQR range 3.1 to 14.6 cm^3^). Post-treatment images were obtained a median of 20 days (range 0 to 92 days) after the completion of chemoradiation and a median of 71 days (range 35 to 159 days) after the pre-treatment imaging scan. The majority of patients received a follow up scan (84%) within one month of completing chemoradiation. The median total tumor volume following chemoradiation was 19.1 cm^3^ (IQR 11.6 to 37.8 cm^3^) and the mean relative change in tumor volume after chemoradiation was -45.8% (IQR -65.3 to 31.0%). The median N2 lymph node volume following chemoradiation was 3.8 cm^3^ (IQR range 1.8 to 7.0 cm^3^) and the median relative change in volume was -44.4% (IQR range -61.7 to -27.8%). The clinical characteristics of age, gender, performance status, histology, overall stage, T stage, type of surgery, and radiation dose were not associated with LRR, PFS, DM, or OS using univariate cox regression (Table 3, **S1 Table**). N3 stage was significantly associated with increased LRR compared to N2 stage (HR 4.66, 95% CI [1.51–14.44], p = 0.01). Multistation nodal disease was associated with decreased PFS (>2 stations vs. 1 station, (HR 2.68, 95% CI [1.06–6.76], p = 0.04) and decreased OS (HR 3.87, 95% CI [1.14–13.18], p = 0.03).

**Table 3:** Univariate analysis of clinical and lymph node imaging characteristics associated with LRR, DM, PFS and OS.

		Locoregional recurrence		Distant Metastasis,		Progression-Free Survival			Overall survival	
**Clinical Characteristics**	n	HR (95% CI)	*p*	HR (95% CI)	*p*		HR (95% CI)	*p*	HR (95% CI)	*p*
Age > 60 (reference age ≤ 60)	38	0.52 (0.21–1.32)	0.17	0.34 (0.16–0.75)	0.01		0.74 (0.41–1.34)	0.32	0.98 (0.49–1.96)	0.95
Sex: Male (reference Female)	22	1.98 (0.81–4.86)	0.13	0.57 (0.23–1.39)	0.22		1.20 (0.64–2.24)	0.56	1.56 (0.77–3.16)	0.21
Performance Status 1–2 (reference 0)	43	1.47 (0.58–3.68)	0.41	0.79 (0.38–1.62)	0.52		0.85 (0.47–1.54)	0.59	0.91 (0.45–1.82)	0.79
Race: White (reference Asian, African-American or Hispanic)	66	0.90 (0.21–3.89)	0.89	3.30 (0.45–24.24)	0.24		0.83 (0.33–2.12)	0.70	0.72 (0.25–2.07)	0.55
Histology: Other (reference Adenocarcinoma)	9	0.72 (0.16–3.18)	0.67	0.38 (0.09–1.63)	0.19		0.40 (0.12–1.34)	0.14	0.38 (0.09–1.60)	0.19
Histology: Squamous cell carcinoma (reference Adenocarcinoma)	16	1.02 (0.33–3.09)	0.98	0.99 (0.40–2.46)	0.99		1.24 (0.61–2.55)	0.55	1.34 (0.59–3.02)	0.49
T Stage										
T2a-T2b (reference T1)	32	0.74 (0.26–2.10)	0.58	0.59 (0.26–1.36)	0.21		0.70 (0.35–1.42)	0.33	0.77 (0.35–1.71)	0.53
T3-T4 (reference T1)	23	0.54 (0.16–1.76)	0.30	0.45 (0.17–1.19)	0.11		0.69 (0.31–1.52)	0.35	0.40 (0.14–1.10)	0.08
N3 stage (reference N2 stage)	7	4.66 (1.51–14.44)	0.01*	1.33 (0.40–4.41)	0.64		2.27 (0.95–5.44)	0.07	2.26 (0.87–5.90)	0.10
Stage IIIB (reference Stage IIIA)	12	2.39 (0.86–6.63)	0.09	1.23 (0.47–3.21)	0.68		2.07 (0.98–4.36)	0.06	1.34 (0.55–3.26)	0.52
Lymph node stations involved (by clinical staging)										
2 stations (reference 1 station)	25	0.24 (0.02–2.65)	0.24	1.22 (0.37–3.98)	0.75		0.85 (0.31–2.38)	0.76	1.55 (0.41–5.91)	0.52
≥3 stations (reference 1 station)	38	4.18 (0.95–18.39)	0.06	1.80 (0.60–5.44)	0.30		2.68 (1.06–6.76)	0.04*	3.87 (1.14–13.18)	0.03*
Radiation > 54 Gy (reference 54 Gy)	27	1.23 (0.51–2.96)	0.65	1.12 (0.54–2.31)	0.75		1.08 (0.59–1.97)	0.81	0.87 (0.42–1.78)	0.70
**Lymph Node Characteristics (continuous)**										
Pre-treatment										
Lymph node volume		1.04 (1.02–1.06)	0.002*	1.00 (0.98–1.03)	0.83		1.03 (1.01–1.04)	0.02*	1.02 (1.00–1.04)	0.07
N2 lymph node volume		1.04 (1.01–1.07)	0.01*	1.00 (0.97–1.03)	0.83		1.03 (1.01–1.05)	0.05	1.02 (1.00–1.04)	0.17
Lymph node to primary tumor ratio		6.11 (1.54–24.18)	0.03*	2.29 (0.66–7.92)	0.71		2.96 (1.08–8.14)	0.09	4.52 (1.43–14.33)	0.07
Post-treatment										
Lymph node volume		1.08 (1.04–1.13)	0.001*	1.01 (0.97–1.06)	0.83		1.05 (1.02–1.09)	0.02*	1.04 (1.01–1.08)	0.07
N2 lymph node volume		1.09 (1.04–1.15)	0.004*	0.99 (0.94–1.06)	0.86		1.05 (1.01–1.10)	0.05	1.04 (1.00–1.09)	0.18
Lymph node to primary tumor ratio		4.96 (1.21–20.40)	0.06	1.92 (0.53–6.92)	0.71		3.00 (1.07–8.40)	0.09	5.48 (1.70–17.62)	0.06

(*) indicates p-values <0.05.

Interestingly, both pre-treatment and post-treatment lymph node volume were associated with locoregional recurrence and progression free survival. Larger pre-treatment lymph node volume was associated with increased LRR (HR 1.04, 95% CI [1.02–1.06], p = 0.002) and decreased PFS (HR 1.03, 95% CI [1.01–1.04], p = 0.02). Increased pre-treatment N2 nodal volume was similarly associated with higher rates of LRR (HR 1.04, 95% CI [1.01–1.07], p = 0.01).

There was a trend toward significance with overall survival as well with larger pre-treatment lymph node volume (HR 1.02, 95% CI [1.00–1.04], p = 0.07) and larger post-treatment lymph node volume (HR 1.04, 95% CI [1.01–1.08], p = 0.07) predicting worse overall survival. Similarly, patients with a large lymph node burden as compared to primary tumor burden also showed a trend toward decreased survival (pre-treatment HR 4.52, 95% CI [1.43–14.33], p = 0.07; post-treatment HR 5.48, 95% CI [1.70–17.62], p = 0.06).

Imaging characteristics were modeled for outcomes of LRR, DM and OS and overall survival using the time dependent concordance index (c-index) as a measure of the quality of the model. Pre-treatment mediastinal lymph node volume had high c-index scores for LRR (0.66, p = 0.01, noether test) (**S1A Fig**) and OS (0.62 respectively, p = 0.04, noether test) (**S1D Fig**) but not DM (**S1B Fig**). Including hilar nodal volumes with mediastinal lymph node volume improved the c-index scores for both LRR (0.71, p<0.001, noether test) and OS (0.67, p = 0.002, noether test). Comparison of lymph node volume as a ratio to the volume of the primary tumor also had a significant association with LRR (CI 0.68, p<0.001, noether test) and OS (CI 0.64, p = 0.01, noether test). Notably, neither pretreatment total tumor volume (inclusive of the primary tumor and lymph nodes) nor primary tumor volume alone were significantly associated with LRR, DM or OS.

Similar to pre-treatment values, post-treatment mediastinal lymph node volume had high c-index scores for LRR (0.72, p = 0.01, noether test) (**S3A Fig**) but not for OS (0.60, p = 0.12). However, including hilar nodal volumes with mediastinal lymph node volume improved the c-index scores for both LRR (0.72, p<0.001, noether test) and OS (0.65, p = 0.01, noether test) (**S3D Fig**). As with pretreatment imaging, the ratio of lymph node volume to the primary tumor volume also had a significant association with LRR (CI 0.65, p<0.001, noether test) and OS (CI 0.66, p = 0.002, noether test). Lymph node volume, primary tumor volume and total tumor volume were not associated with DM (**S3B Fig**).

### Multivariate analysis

Multiple imaging characteristics were significantly associated with LRR, PFS and OS on univariate Cox regression. In order to determine the optimal parameter(s), all imaging covariates were entered into a lasso variable selection and k-fold cross validation. Cross validation demonstrated an association between post-treatment lymph node volume and LRR and PFS. Similarly there was an association between post-treatment lymph node volume as a ratio to the primary tumor volume and OS. Despite a strong univariate association and significance using the Kaplan Meier estimate (**S2 and S4 Figs**), mediastinal nodal volume was not selected in the final cross-validated model for any outcome. The resulting variables were then entered into a multivariable Cox regression with the clinical characteristics previously found to be significant on univariate analysis including clinical N stage and number of positive lymph node stations **(Table 4)**. On multivariate analysis, post-treatment lymph node volume remained independently associated with LRR (HR 1.07, 95% CI [1.02–1.13], p = 0.01) but was not associated with progression free survival (p = 0.16). Interestingly, while post-treatment lymph node volume was not selected in the cross-validated model, the ratio of post-treatment lymph node volume to primary tumor volume was a significant predictor of overall survival (HR 3.68, 95% CI [1.09–12.47], p = 0.04), suggesting that patients with larger post-treatment lymph node volumes were more likely to have increased locoregional recurrence and decreased survival.

**Table 4:** Multivariate analysis of clinical and lymph node imaging characteristics associated with LRR, PFS and OS. (*) indicates p-values <0.05.

	Locoregional recurrence		Progression-free survival		Overall Survival	
	HR (95% CI)	*p*	HR (95% CI)	*p*	HR (95% CI)	*p*
N3 Stage (reference N2 Stage)	1.58 (0.33–7.47)	0.57				
Lymph node station >3 (reference 1 station)			2.07 (0.76–5.68)	0.16	2.83 (0.80–10.05)	0.11
Post-treatment lymph node volume (per cm^3^)	1.07 (1.02–1.13)	0.01*	1.03 (0.99–1.07)	0.16		
Post-treatment lymph node volume to primary tumor volume ratio					3.68 (1.09–12.47)	0.04*

**Fig 1:**
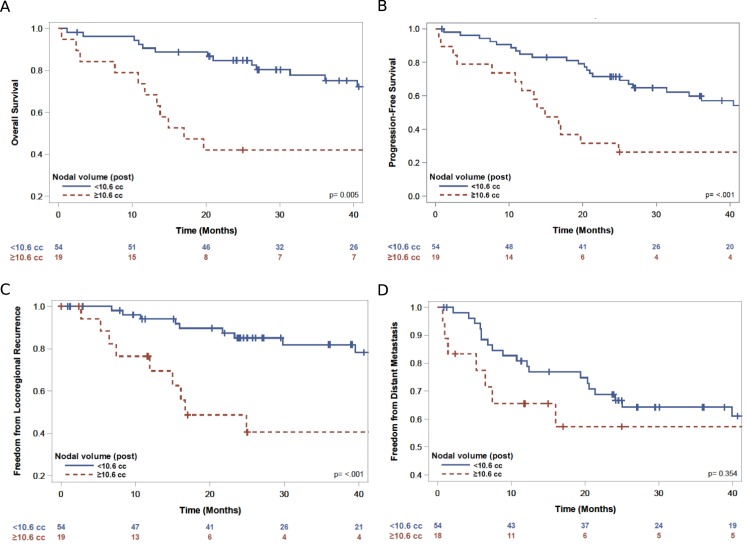
**Kaplan Meier curves for nodal volume following chemoradiation grouped by upper quartile (>10.6 cm^3^) vs lower three quartiles (<10.6 cm^3^) for (a) OS (3 year survival 78% vs. 42%, p = 0.005), (b) PFS (3 year PFS 59% vs. 26%, p<0.001), (c), LRR (3 year freedom from LRR 82% vs. 41%, p<0.001), (d) DM (3 year freedom from DM 64% vs. 57%, p = 0.35)**.

Kaplan-Meier analysis demonstrated that patients with lymph node volume greater than 10.6 cm^3^ after the completion of preoperative chemoradiation, (representing the upper quartile of post-treatment volumes), had increased LRR (p<0.001) (**Fig 1C**) and decreased OS (p = 0.005) compared to patients with lymph node volume less than 10.6 cm^3^ (**Fig 1A, S5 Fig, Table 5**).

**Table 5:** Treatment outcomes reported for median time to event and 3 year Kaplan-Meier estimate for small lymph node volume (quartiles 1–3) versus large lymph node volume (quartile 4) following chemoradiation.

	Median time to event (months)		3 year event estimate		p
	Quartile 1–3	Quartile 4	Quartile 1–3	Quartile 4	
Overall survival	80.0	17.0	78%	42%	0.005
Progression free survival	60.5	14.9	59%	26%	<0.001
Freedom from locoregional recurrence	NR	16.7	82%	41%	p<0.001
Freedom from distant metastasis	69.2	62.9	64%	57%	p = 0.35

To complete the analysis of imaging predictors of outcome, we additionally investigated the association between survival and the change in tumor volume between pretreatment and post-treatment CT imaging. However, there were no significant associations between tumor volume change or lymph node volume change and LRR, PFS, DM, or OS. Since tumor response in clinical trials is assessed using the diameter of tumors based on RECIST criteria, RECIST response was also compared with outcomes. However, there was no association between RECIST response and LRR, DM, PFS or OS (**S1 Table**).

### Lymph node volume does not predict nodal downstaging

Since lymph node volume was predictive of LRR and OS, we investigated the relationship between lymph node burden and the endpoint of nodal downstaging. Forty-seven patients (64%) were downstaged to either ypN1 or ypN0 by pathologic staging at the time of surgery. Forty patients (54.8%) achieved complete nodal clearance to ypN0. Nodal downstaging itself was not associated with LRR, DR or OS, however, complete nodal clearance was associated with decreased LRR (HR 0.97, 95% CI [0.40–0.98], p = 0.04).

Using univariate logistic regression, there was no significant association between pre-treatment N2 volume (p = 0.62), post-treatment N2 volume (p = 0.84), or change in volume (p = 0.50) and nodal downstaging. Additionally, there was no association between downstaging and total pre-treatment volume (p = 0.74), total post-treatment volume (p = 0.42), or change in total tumor volume (p = 0.47). Similarly, there were no significant associations between N2 volumes and nodal clearance. With respect to clinical features, there were no associations between age, race, gender, performance status, number of positive lymph node stations, radiation dose, or clinical stage and nodal downstaging or clearance.

## Discussion

Stage III NSCLC is a broad category composed of heterogeneous tumor populations. Current TNM staging accounts for anatomic lymph node involvement but does not incorporate the significant variation in tumor burden of involved lymph nodes [22]. Multiple studies have shown that mediastinal nodal clearance is a strong predictor of OS suggesting that nodal tumor burden, in addition to anatomic involvement, is an important prognostic marker of survival [5–10]. These studies suggest a clear link between local disease control and survival. Additional methods for patient stratification by tumor burden are thus relevant for guiding clinical decision-making in patients with locally advanced NSCLC.

In this study, we demonstrate the correlation between involved lymph node tumor volume and local control as well as OS in patients with mediastinal nodal involvement. We demonstrate that larger lymph node tumor burden prior to chemoradiation, as determined by number clinically positive nodal stations or lymph node volume, is strongly associated with locoregional recurrence and overall survival. Although N stage, nodal stations and nodal volume are similar measures of disease burden, we additionally demonstrate that nodal volume is an independent predictor of LRR from N stage. For patients with multiple stations of mediastinal nodal involvement, larger lymph node volume was associated with increased locoregional recurrence whereas nodal stations were not. Furthermore, our results demonstrate the unique finding that patients with large lymph node burden prior to surgical resection, even following chemoradiation, are at high risk for local recurrence and decreased survival. In agreement with these results, a greater lymph node volume relative to primary tumor volume ratio was also associated with decreased OS on multivariate analysis. These results have implications for clinical tumor response assessment as they can be utilized to demonstrate the potential efficacy of chemoradiation therapy prior to surgery.

Our results complement the results of Alexander et al who previously reported an association between larger pre-treatment lymph node volume and decreased overall survival in patients treated with definitive chemoradiation [15]. However, other definitive chemoradiation studies have reported a lack of association between pre-treatment lymph node volume and survival. Dehing-Oberije et al and Basaki et al report no association between pre-treatment nodal volume and survival [13,16]. Such differences may be partially explained by heterogeneity in treatment modalities, tumor volume thresholds and segmentation used in these studies. Dehing-Oberjie et al report a much larger greater nodal mean GTV of 32 cm^3^ in their study, in contrast to our mean of 15.7 cm^3^ (median 10.3 cm^3^). The larger nodal volume in their study may reflect greater lymph node involvement in their patient population or expansion of the GTV to include surrounding normal tissue. Basaki et al use a cutoff of 15 cm^3^ for total nodal volume, which is comparable to our study, however their analysis also included patients with N1 disease and was limited to patients undergoing definitive radiation therapy.

Despite these different findings on the prognostic ability of pre-treatment lymph node volume in prior reports, our results are unique in that to our knowledge this is the first report of the prognostic significance of post-treatment lymph node volume following neoadjuvant chemoradiation and prior to resection. The aforementioned studies reported the prognostic significance or lack thereof for lymph node volume only prior to the initiation of chemoradiation. Stinchcombe et al described the association between larger post-chemotherapy tumor volume in stage IIIA-IIIB patients and decreased survival but did not separately analyze lymph node or mediastinal nodal volume [23]. While, we did not find an association between larger total tumor volumes (inclusive of the primary tumor), our results demonstrate that lymph node volume is an important marker of survival. This study provides an additional time point for clinical decision making by analyzing post-neoadjuvant therapy nodal volume immediately prior to surgery, suggesting that patients with both large pre- and post-treatment volume lymph node involvement have increased risk of locoregional recurrence and decreased progression-free and overall survival. These results are consistent with prior studies that report residual disease as a prognostic factor for LRR [24], as well as new staging guidelines that account for nodal station involvement [25].

Of note, the change in lymph node volume was not prognostic for survival, suggesting that absolute lymph node volume is more relevant for patient stratification. Furthermore, we provide evidence for the lack of histopathologic correlation with lymph node volume, suggesting that even significant changes in volume or diameter of the involved lymph nodes do not necessarily have pathologic correlation.

The findings of this study must be interpreted in the context of potential limitations including the retrospective nature of the study and small sample size. First, patients in our cohort had longer overall survival than has been previously reported. Our median survival of 78 months is considerably longer than previously reported median survival rates of 19–26 months for patients receiving chemoradiation and surgical resection [10,26,27]. As such, we acknowledge that this group of patients was likely selected to undergo surgical resection due to better tolerance and/or response to preoperative chemoradiation than patients who did not receive surgical resection. This may reduce generalizability to other LA NSCLC patients. Second, given the small sample size, this study may be underpowered to detect associations between nodal downstaging and imaging characteristics. These findings will require validation in larger cohorts. Furthermore, given that CT imaging was used for treatment monitoring in this study, it is not clear how these results will apply to other imaging modalities such as FDG-PET which have previously been associated with clinical outcomes [28,29]. Despite these limitations, the results of this study demonstrate that nodal volume measured before and after preoperative chemoradiation has significant prognostic value for local control and overall survival, even within a selected trimodality population. We identify patients at high risk of local recurrence based on lymph node volume following neoadjuvant therapy, which could be used to identify candidates for additional cycles of chemotherapy or radiation dose escalation. However, we acknowledge that the use of additional therapy for higher risk patients has yet to be prospectively studied.

## Conclusion

This study demonstrates the association between lymph node size and clinical outcomes of LRR and OS before and after neoadjuvant chemoradiation for patients undergoing trimodality therapy. This data adds to the growing body of literature underscoring the importance of patient stratification using additional imaging parameters beyond TNM staging. For patients with locally advanced NSCLC, nodal size during the course of chemoradiation should thus be incorporated along with clinical characteristics to guide clinical decision-making.

## Supporting information

S1 Fig **Comparison of univariate concordance index (c-index) values of pretreatment imaging characteristics for LRR (A), DM (B) and OS (C) grouped by location of measurement.** (*) indicates p-values <0.05 using noether test from random (CI 0.5).
(PDF)

S2 Fig **Kaplan Meier curves for N2 nodal volume prior to chemoradiation grouped by upper quartile (>14.6 cm3) vs lower three quartiles (<14.6 cm3) for LRR (a) and OS (b).** + marks represent censored results. P-values calculated using log-rank test. p<0.05 considered significant.
(PDF)

S3 Fig **Comparison of univariate concordance index (c-index) values of posttreatment imaging characteristics for LRR (a), DR (b), and OS (c) grouped by volume and diameter measurements**. (*) indicates p-values <0.05.
(PDF)

S4 Fig **Kaplan Meier curves for residual N2 nodal volume following chemoradiation grouped by upper quartile (>7.0 cm3) vs lower three quartiles (<7.0 cm3) for LRR (a) and OS (b).** + marks represent censored results. P-values calculated using log-rank test. p<0.05 considered significant.
(PDF)

S5 Fig Kaplan Meier curves for post-treatment nodal volume following chemoradiation grouped by quartile for freedom from locoregional recurrence. + marks represent censored results. P-values calculated using log-rank test. p<0.05 considered significant.
(PDF)

S1 Table Univariate and multivariate analysis. (*) indicates p-values <0.05.
(XLSX)

S1 Dataset Supporting dataset. (XLS)
