# Supplementary figures and images for: Lymph node volume predicts survival but not nodal clearance in Stage IIIA-IIIB NSCLC
Source: PLoS One. 2017 Apr 20;12(4):e0174268. doi: 10.1371/journal.pone.0174268 (PMC5398511; doi:10.1371/journal.pone.0174268)

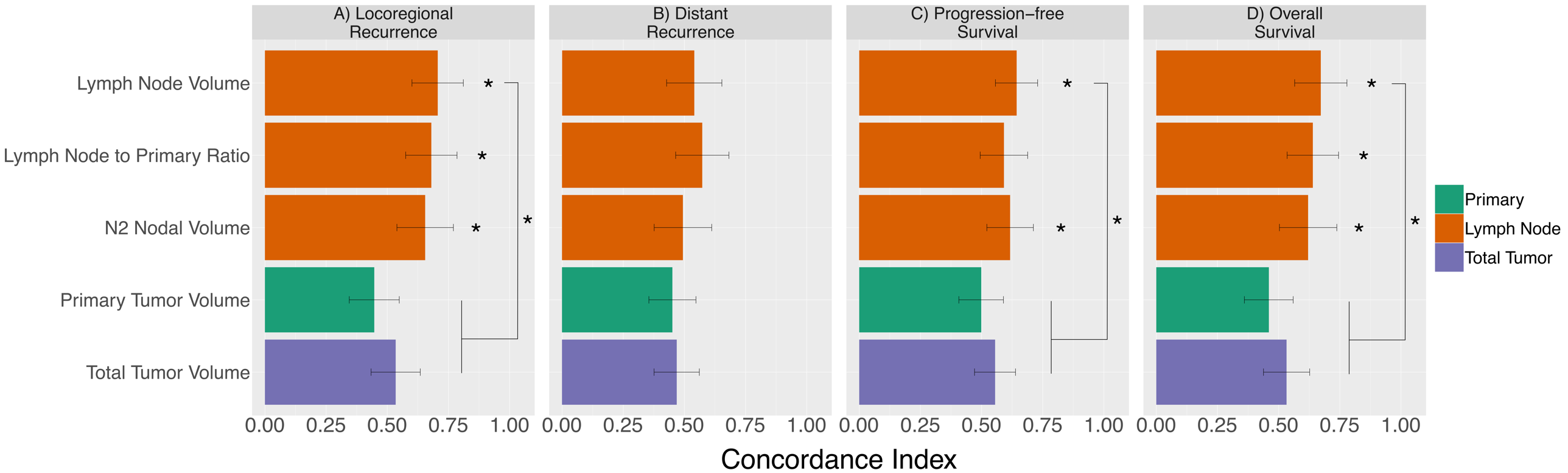

Supplement: S1 Fig — Comparison of univariate concordance index (c-index) values of pretreatment imaging characteristics for LRR (A), DM (B) and OS (C) grouped by location of measurement. (*) indicates p-values <0.05 using noether test from random (CI 0.5). (PDF) [file pone.0174268.s001.pdf]

a

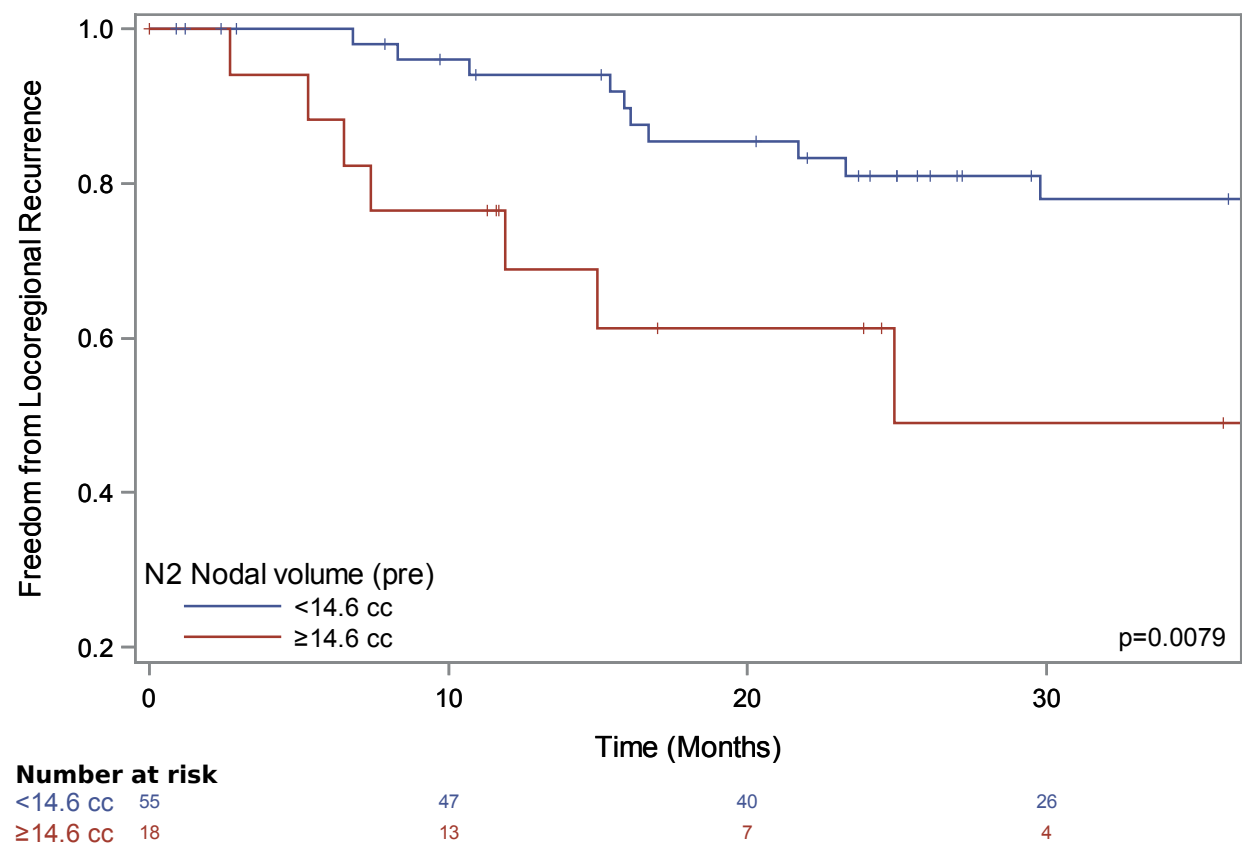

b

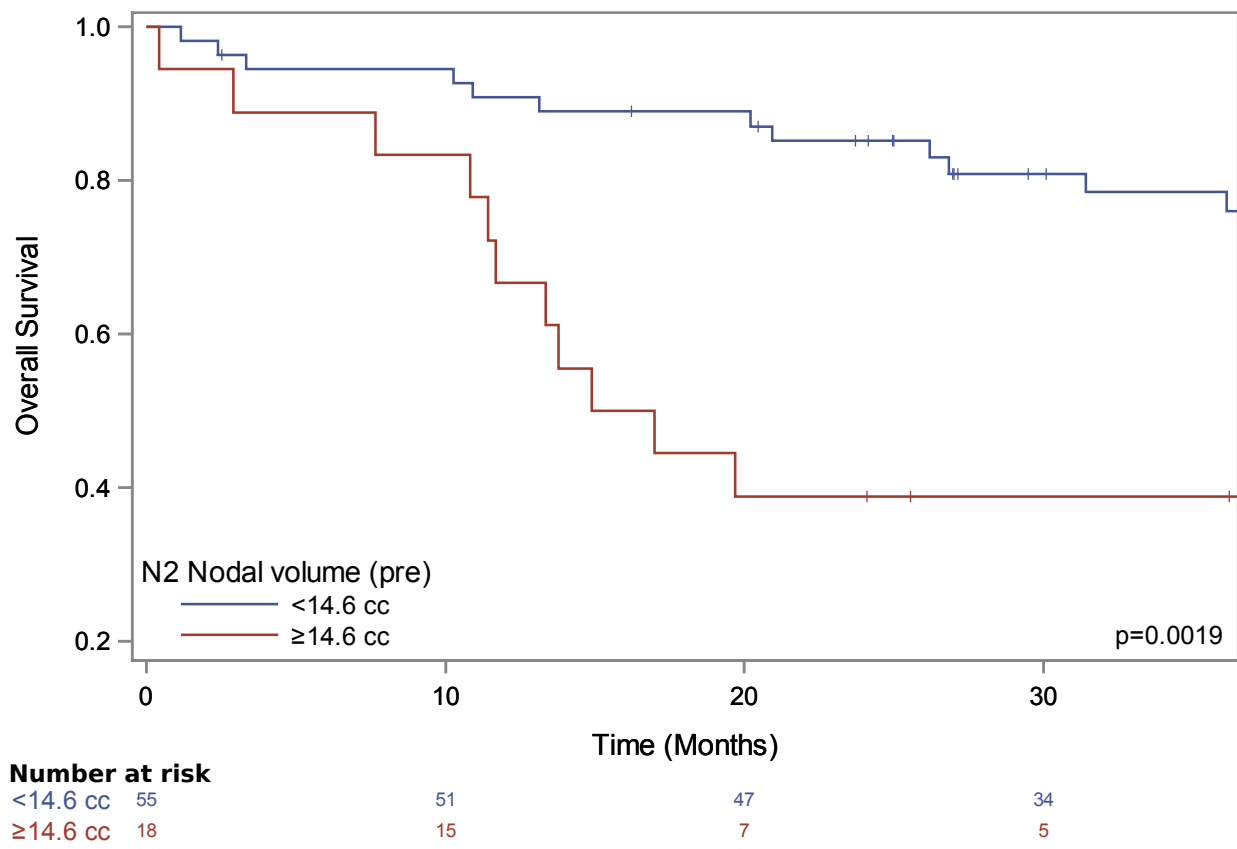

Supplement: S2 Fig — Kaplan Meier curves for N2 nodal volume prior to chemoradiation grouped by upper quartile (>14.6 cm3) vs lower three quartiles (<14.6 cm3) for LRR (a) and OS (b). + marks represent censored results. P-values calculated using log-rank test. p<0.05 considered significant. (PDF) [file pone.0174268.s002.pdf]

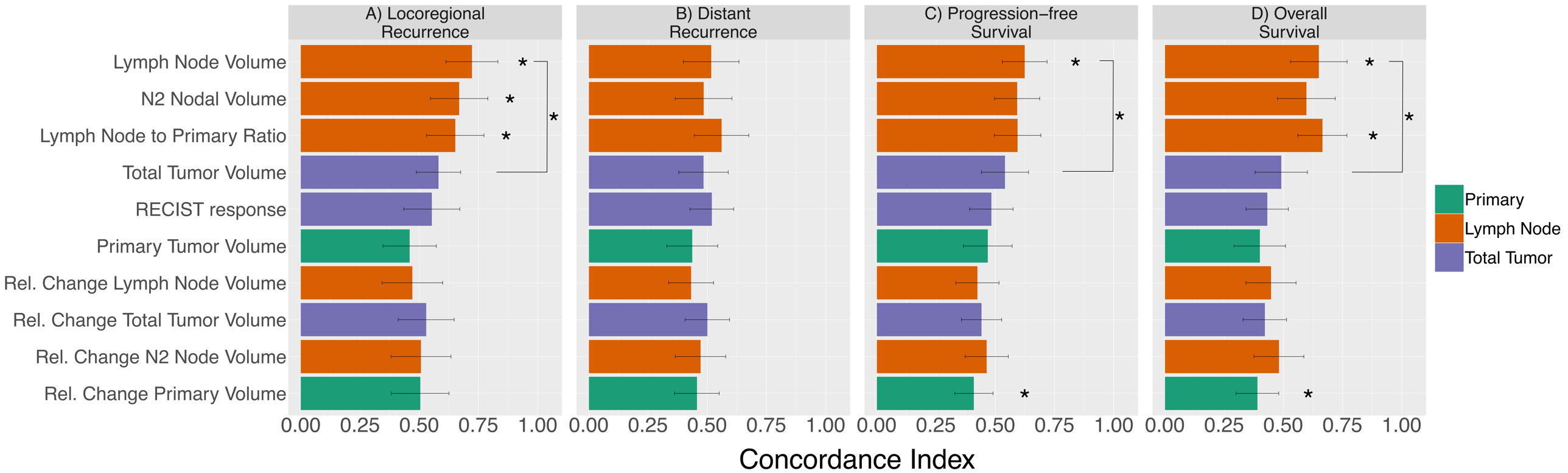

Supplement: S3 Fig — Comparison of univariate concordance index (c-index) values of posttreatment imaging characteristics for LRR (a), DR (b), and OS (c) grouped by volume and diameter measurements. (*) indicates p-values <0.05. (PDF) [file pone.0174268.s003.pdf]

a

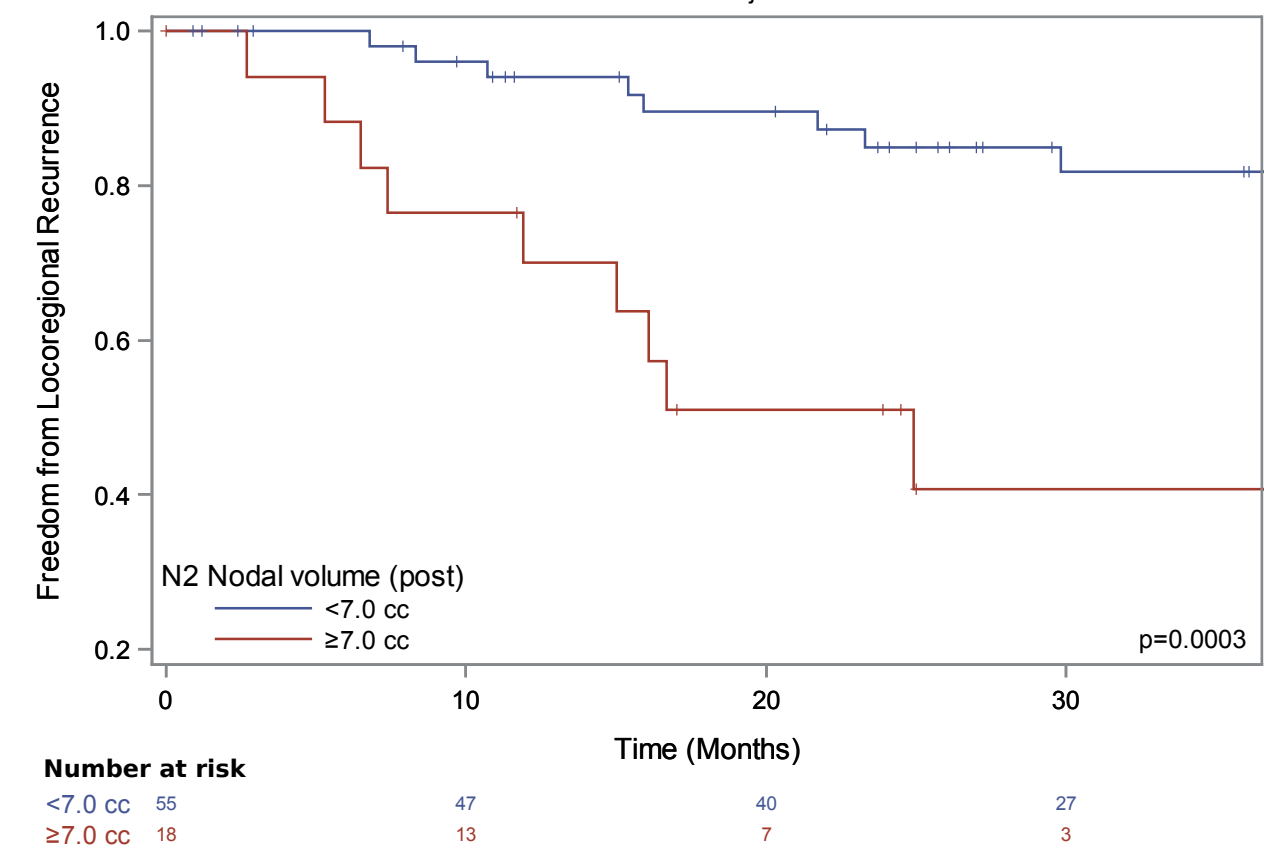

b

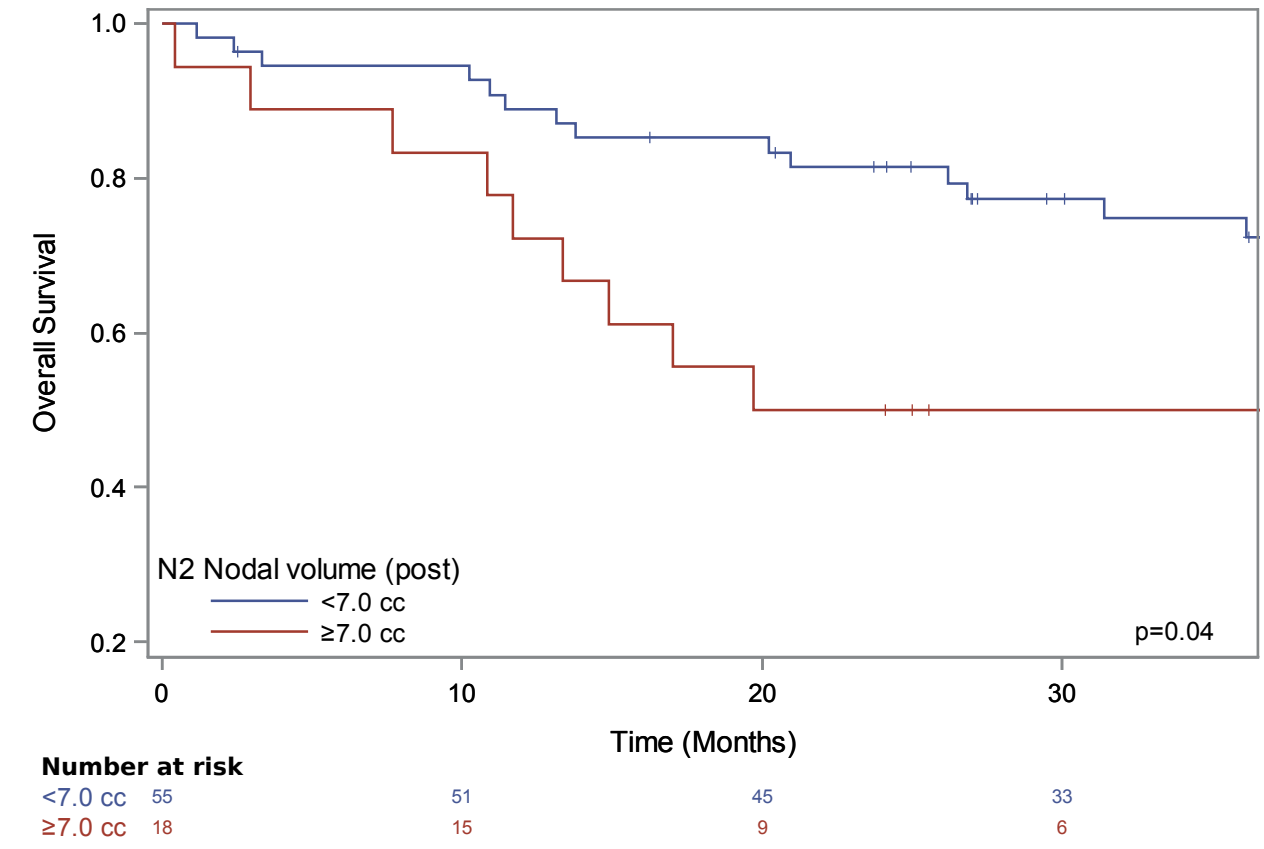

Supplement: S4 Fig — Kaplan Meier curves for residual N2 nodal volume following chemoradiation grouped by upper quartile (>7.0 cm3) vs lower three quartiles (<7.0 cm3) for LRR (a) and OS (b). + marks represent censored results. P-values calculated using log-rank test. p<0.05 considered significant. (PDF) [file pone.0174268.s004.pdf]

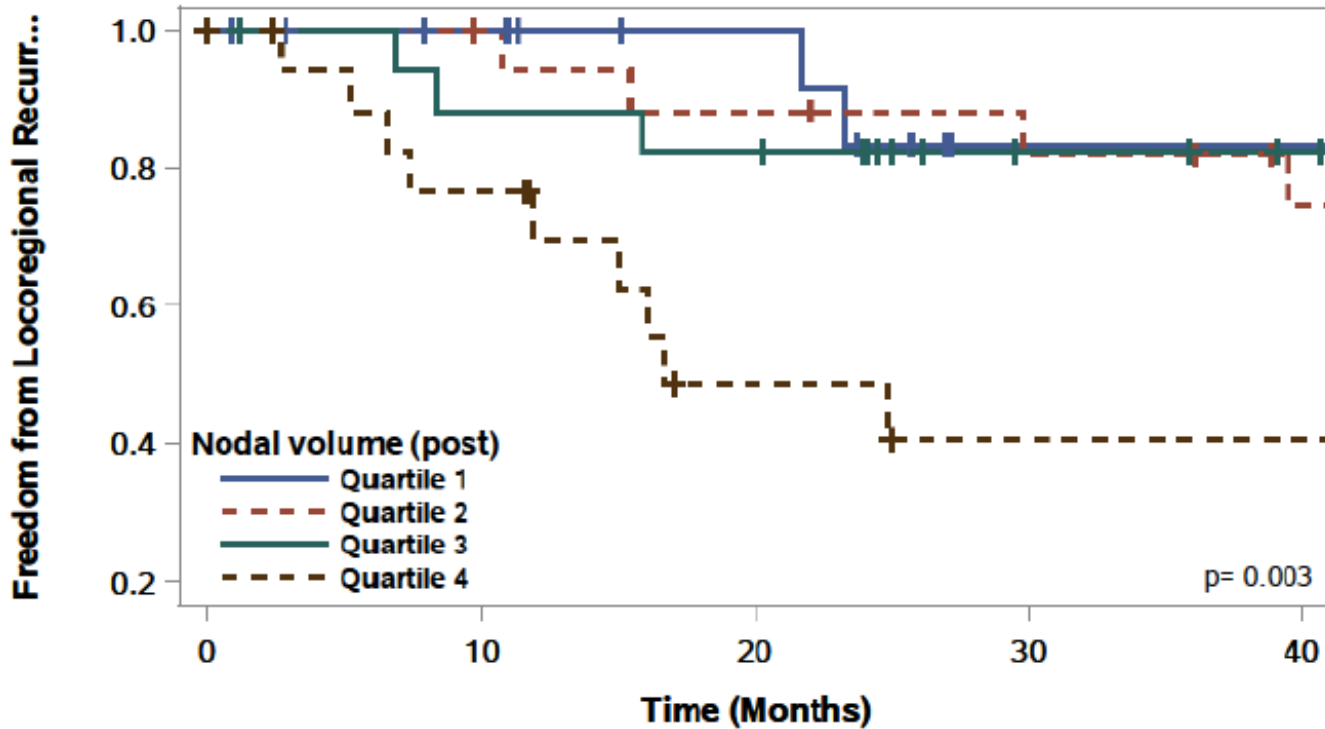

|            |    |    |    |    |    |
|------------|----|----|----|----|----|
| Quartile 1 | 18 | 15 | 12 | 6  | 6  |
| Quartile 2 | 18 | 17 | 15 | 13 | 10 |
| Quartile 3 | 18 | 15 | 14 | 7  | 5  |
| Quartile 4 | 19 | 13 | 6  | 4  | 4  |

Supplement: S5 Fig — + marks represent censored results. P-values calculated using log-rank test. p<0.05 considered significant. (PDF) [file pone.0174268.s005.pdf]
